# Regimental Camp Sanitary Reports

**Published:** 1911-03-25

**Authors:** 


### THE ROYAL ARMY MEDICAL CORPS SECTION.

### REGIMENTAL CAMP SANITARY REPORTS.

THE practice followed in some of the commands of encouraging the regimental medical officers to send in sanitary reports on the camp with which they have been associated is an excellent procedure and one calculated to bring under official notice many points of detail that would otherwise escape observation during an ordinary inspection. Such reports, too, should be instrumental in helping on the solution of several problems that have not yet been satisfactorily solved, especially in matters connected with the conservancy of camps; and by their means there may be conveyed to those in authority observations on the system at present followed and useful suggestions for the improvement of the working of future camps. In view of their importance, and to make such reports of value they should be comprehensive, and they should be drawn up to embrace the following points:—(1) General detail; (2) water supply; (3) food supply; (4) conservancy of the camp; (5) health of the men; and (6) special remarks.

General details include the site of the camp, its size, the nature of its soil, its approaches from the main road, the nearest railway station, and the facilities for field training in its neighbourhood. In addition to this there should be further stated the length of time for which the camp was held and the number of men actually present in it. Special reference, too, should be made to the number and efficiency of the regimental stretcher-bearers and sanitary men attending camp, for their duties are very important, and there are not always given by the commanding officers of the units sufficient facilities for their training. The system now in vogue of making the bandsmen of the unit act as stretcher-bearers has its objections, and it has not proved altogether a success. The regimental medical officer should also put on record the nature and amount of instruction given by him during camp, and the regularity or otherwise of the delivery of the camp equipment.

As regards the water supply, it is usual to have this tested and its purity certified before the locality of the camp is fixed on, but the supply of a sufficient amount of wholesome water is such an absolute sanitary necessity that it should receive special attention, and every camp report should speak definitely as to its quality and quantity. The same remarks apply to the food supply. Too much attention cannot be paid by the regimental medical officer to the rations furnished to his men and to the methods employed in cooking them. He should satisfy himself that they are of good quality, sufficiently nourishing, capable of variation, and properly prepared; any defects on these points should be specially noted and reported on. The daily inspection of the meat should ensure the quality being good; but as meat is handed over in the afternoon previous to the day on which it is used, arrangements should be made for its storage in a proper place until it is issued to the different regiments. Another point to be remembered is that a good deal of the meat provided in camps is frozen, and if it is not thoroughly thawed before roasting it is served up to the men in a raw and unpalatable condition, with the result that it is not eaten, but thrown aside and finds its way into the rubbish barrels. The thawed condition may be satisfactorily overcome by stewing the meat, but this necessitates stewed meat being given day after day to the men monotonously. The solution of the difficulty is to have the frozen meat properly thawed in an "Aldershot oven" for an hour or so before cooking, and then it can be easily dealt with by roasting or any other method. In a camp report all new cooking arrangements should be reported on. Something more is needed than the Soyer's stoves and camp-kettles supplied by the authorities. It is impossible to give with these the variation in meals that is needed. Consequently the different units furnish themselves with various cooking stoves, some of them of undoubted utility, and to these attention should be drawn. Regimental medical officers should satisfy themselves, too, that the cooks are efficient and thoroughly instructed, because it is only with such properly-trained men that economy can be secured and wastage prevented. Other essentials are a proper location of the cookhouses, great cleanliness in connection with the preparation of food, ample facilities for the washing of the hands of the cooks and assistants, a plentiful supply, if possible, of hot water for the cleansing of dishes and plates, care being taken that this last is not carried out in any of the ablution benches. Upon all these points it is important that it should be possible to give a satisfactory report.

Passing now to the conservancy of the camp, the regimental medical officer will have to deal in his report with the form of latrines used, with the disposal of refuse and rubbish, with the washing-places, the cookhouses, and the horse lines. All of these demand the greatest care, as all of them are the source of soil pollution, and to prevent this every care must be taken. From some of them issue greasy and dirty water which thrown on the ground soon emits offensive odours and attracts flies, so that its disposal must be provided for and some form of "grease trap" devised. Full information should be furnished as to how all these various matters have been dealt with and individual experience recorded. Opinion is yet divided as to whether one long trench or a succession of short trenches is preferable for the latrines of a temporary camp. Nor does unanimity exist as to the best form of destructor to be used for the burning of the garbage and refuse of the camp, so that there is room for ingenuity and commonsense in devising an arrangement for this end, and any new plan adopted should be fully described, and an opinion expressed thereon.

Every regimental report must, of course, deal with the health of the individual unit; serious cases of illness must be fully gone into. It is, however, under the heading of special remarks that the regimental surgeon has an opportunity of expressing his views on many points of interest and importance, and of this he should avail himself. There is no doubt that in many details connected with our Territorial camps modifications are needed. Thus, there is a very general feeling that the medical equipment as at present issued to regiments is deficient in such matters as aperients, foot-powders, face-lotions, and dressings, and an expression of opinion to that effect from the various regimental surgeons would prove helpful in having this remedied. One lesson, too, that camp-life teaches and that the medical officers of the regiment should take to heart, is that each unit should have at least two lectures on sanitation before going to camp, and that attendance on them should be compulsory, as ignorance and not carelessness is often the explanation of the misuse of the various sanitary arrangements in use in camp. In the same way notices explaining the use of latrines, destructors, and refuse pits would be of very great assistance, and would help the sanitary squads in their work. It is not easy briefly to specify all the matters that should come under the observation of a regimental medical officer during a camping period, but the fact that he is asked to embody his experience in a report is in itself a powerful incentive to him to carry on his work with increased care and observation.

